# Follicular fluid-derived exosomal LINC02701 promotes granulosa cell apoptosis through the GRP75–P53 axis in active endometriosis

**DOI:** 10.3389/fcell.2026.1805254

**Published:** 2026-05-22

**Authors:** Feng Zhou, Xinnan Tang, Kaiquan Li, Nan Lu, Yundong Mao

**Affiliations:** 1 Clinical Center of Reproductive Medicine, The First Affiliated Hospital with Nanjing Medical University/Jiangsu Province Hospital, Nanjing, China; 2 State Key Laboratory of Reproductive Medicine and Offspring Health, Nanjing Medical University, Nanjing, China

**Keywords:** endometriosis, exosomes, granulosa cells, GRP75–P53 axis, LINC02701

## Abstract

**Background:**

Endometriosis (EMs)-associated infertility has been linked to alterations in the follicular microenvironment; however, the role of exosomal long non-coding RNAs (lncRNAs) in granulosa cell dysfunction remains incompletely understood.

**Methods:**

Follicular fluid-derived exosomes were isolated from patients with active endometriosis (EMs_A), controlled endometriosis (EMs_C), and non-endometriosis controls. Transcriptomic sequencing was performed to identify differentially expressed exosomal lncRNAs. Functional assays, loss-of-function experiments, mechanistic analyses, and pharmacological inhibition studies were conducted in granulosa cells.

**Results:**

LINC02701 was identified as one of the most significantly enriched exosomal lncRNAs in active endometriosis. Functional assays demonstrated that LINC02701 can be transferred into granulosa cells, where it suppresses proliferation and promotes apoptosis. Knockdown of LINC02701 attenuated granulosa cell apoptosis, whereas pharmacological inhibition of P53 partially reversed the pro-apoptotic effects induced by LINC02701. Mechanistically, LINC02701 directly interacted with GRP75, disrupted the GRP75–P53 interaction, enhanced nuclear accumulation of P53, and activated downstream apoptotic signaling, including BAX and PUMA. Clinically, EMs_A patients exhibited elevated CA125 levels, increased uterosacral ligament tenderness, and reduced Day-3 high-quality embryo rates, which were consistent with the observed cellular phenotypes.

**Conclusion:**

These findings suggest that exosomal LINC02701 may contribute to granulosa cell dysfunction through modulation of the GRP75–P53 axis and may be associated with impaired early embryo quality in active endometriosis, highlighting its potential as a candidate biomarker for EMs-associated infertility.

## 1 Introduction

Endometriosis (EMs) is a chronic inflammatory disease affecting approximately 10% of reproductive-aged women, and its association with infertility is well established (As-Sanie et al., 2025; Bulun et al., 2019). Women with EMs exhibit a two-to four-fold increase in infertility risk, which has been partially attributed to adverse reproductive outcomes (Fan et al., 2025; Leone Roberti Maggiore et al., 2024). This impairment is thought to arise, at least in part, from inflammatory and endocrine disturbances that reshape the follicular microenvironment and disrupt the conditions required for optimal oocyte maturation (Dumesic et al., 2015; Gupta et al., 2008).

Because oocyte development depends heavily on the biochemical and paracrine interactions within follicular fluid, alterations in its composition have been closely linked to ovarian dysfunction in reproductive diseases (Di, 2016; Zhang et al., 2020). Extracellular vesicles, particularly exosomes, constitute an important regulatory component of this microenvironment. These vesicles transfer proteins, lipids, and non-coding RNAs among follicular cells, thereby modulating proliferation, metabolic activity, and oocyte-related developmental processes (Kowalczyk et al., 2022; Zhang et al., 2021). Perturbations in exosomal cargo have been implicated in multiple reproductive pathologies and are increasingly recognized as sensitive indicators of microenvironmental dysregulation (Chen and Wang, 2022; Esfandyari et al., 2021; Freger et al., 2021; Machtinger et al., 2016).

Our preliminary work demonstrated that exosomes from patients with active endometriosis are internalized by granulosa cells, where they induce oxidative stress, mitochondrial dysfunction, reduced proliferation, and apoptosis—processes tightly associated with adverse reproductive outcomes (Zhang et al., 2024a). These findings suggest that abnormalities in exosomal cargo may represent a mechanistic link between active EMs and adverse reproductive outcomes. Among such cargoes, non-coding RNAs, particularly long non-coding RNAs (lncRNAs), are emerging as important regulators of cellular signaling, epigenetic modification, and stress responses (Chen and Kim, 2024; Li et al., 2021). Dysregulated exosomal lncRNAs have been reported in reproductive disorders such as polycystic ovary syndrome (Xin et al., 2024; Yu et al., 2024), yet their contribution to EMs-associated granulosa cell dysfunction remains largely unexplored.

Through transcriptomic profiling of follicular fluid-derived exosomes, we identified LINC02701 as a disease activity-associated lncRNA that is markedly enriched in the EMs_A group. Granulosa cells, as major recipients of follicular exosomes, are essential for metabolic support and meiotic regulation of the oocyte; their apoptotic loss has been associated with diminished embryo quality and poor assisted reproductive technology (ART) outcomes. We therefore hypothesized that exosomal LINC02701 contributes to granulosa cell dysfunction in EMs by altering key protein-interaction networks that govern cell survival.

This study was designed to: (1) evaluate LINC02701 expression in exosomes across EMs patient subgroups and examine associations with clinical disease indicators and embryo quality; (2) determine the functional effects of LINC02701 on granulosa cell proliferation and apoptosis; (3) elucidate the underlying molecular mechanism, with a particular focus on the GRP75–P53 regulatory axis; (4) explore the potential of LINC02701 as a candidate biomarker and mechanistically relevant regulator in EMs-associated infertility. Collectively, these analyses provide a mechanistic framework linking exosomal lncRNA dysregulation to granulosa cell injury and impaired early embryo quality in active endometriosis.

## 2 Materials and methods

### 2.1 Study population and clinical grouping

A total of 24 patients undergoing *in vitro* fertilization (IVF) at the First Affiliated Hospital with Nanjing Medical University/Jiangsu Province Hospital/Jiangsu Women and Children Health Hospital between April and August 2024 were enrolled, with all participants aged ≤35 years and meeting the inclusion criteria. Patients assigned to the active endometriosis group (EMs_A) satisfied the following criteria: (I) laparoscopic diagnosis of endometriosis with current manifestation of ≥1 of the following: serum CA125 ≥15 U/mL, visual analog scale (VAS) for uterosacral ligament tenderness >4, or recurrence of dyspareunia/dysmenorrhea; (II) presence of ≥3 of five symptoms (infertility, dysmenorrhea, dyspareunia, uterosacral ligament tenderness VAS >4, or CA125 ≥15U/mL) (Zhang et al., 2024b). Patients assigned to the controlled endometriosis group (EMs_C) required laparoscopic diagnosis/excision of endometriotic lesions plus current uterosacral ligament tenderness VAS ≤4 and CA125 <15 U/mL, with GnRH agonist use permitted. The non-endometriosis control group (Ctrl) comprised patients with male/tubal factor infertility, absence of dysmenorrhea/dyspareunia/uterosacral ligament tenderness, and CA125 levels <15 U/mL, similarly restricted to GnRH-A or mild protocols. The exclusion criteria applicable to all groups included the presence of ovarian cysts, diminished ovarian reserve (AMH<1.1 ng/mL and/or AFC<7), polycystic ovary syndrome, and endocrine/metabolic disorders. Because ovarian stimulation protocols may influence the follicular microenvironment, protocol information was collected and considered during interpretation of exosomal cargo-related findings. Given the exploratory nature of this study and the strict enrollment criteria, the clinical cohort was intended primarily for mechanistic association analysis rather than formal biomarker model development. Written informed consent was obtained from all participants, and ethical approval was granted (No. 2013-SRFA-093).

### 2.2 Follicular fluid collection and exosome isolation

Follicular fluid (FF) was collected from follicles ≥14 mm on the day of oocyte retrieval. To eliminate contaminating cells and debris, the samples underwent differential centrifugation at 600 *g* for 10 min, 2,000 × g for 10 min, and 12,000 × g for 30 min at 4 °C. The supernatants were filtered through 0.22 μm membranes and stored at −80 °C. Exosomes were isolated by ultracentrifugation at 120,000 × g for 3 h at 4 °C. The pellets were resuspended in PBS and characterized as follows: transmission electron microscopy (TEM) to assess morphology, nanoparticle tracking analysis (NTA) to determine particle size distribution in the Ctrl, EMs_C, and EMs_A groups, and Western blotting using the input follicular fluid (Input FF), exosome pellet (Exo), and exosome-depleted supernatant (Sup) fractions. Canonical exosomal markers (CD9, CD63, and TSG101) and the endoplasmic reticulum marker Calnexin were detected to assess vesicle identity and potential contamination. These analyses were performed to strengthen extracellular vesicle characterization in line with MISEV2018 recommendations (Théry et al., 2018).

### 2.3 High-throughput sequencing and identification of differentially expressed lncRNAs

To identify exosomal lncRNAs associated with endometriosis activity, FF-derived exosomes from EMs_A, EMs_C, and Ctrl groups were subjected to RNA sequencing. Libraries were sequenced on the DNBSEQ platform using a paired-end 100-bp (PE100) strategy. After quality filtering, approximately 11.46 Gb of clean data were obtained per sample, corresponding to approximately 119–121 million clean reads per sample. Clean reads were aligned to the human reference genome (NCBI GCF_000001405.39_GRCh38.p13) using HISAT, and aligned to reference genes using Bowtie2. The specific library preparation kit/type and lncRNA annotation database were not specified in the provider brief report. Differential expression was analyzed across three pairwise comparisons: EMs_A vs. Ctrl, EMs_C vs. Ctrl, and EMs_A vs. EMs_C. lncRNAs with |log_2_FC| > 2 and FDR <0.05 were considered significantly differentially expressed. Candidate lncRNAs were prioritized based on three criteria: (1) significant upregulation in EMs_A vs. Ctrl, (2) reduction in EMs_C vs. EMs_A (disease-activity responsiveness), (3) no significant elevation in EMs_C vs. Ctrl (exclusion of nonspecific markers). The top candidates were validated by quantitative PCR (qPCR).

### 2.4 RNase protection assay

To assess whether LINC02701 was enclosed within membrane-bound extracellular vesicles, an RNase protection assay was performed on purified vesicle preparations. Equal amounts of vesicles were allocated to each treatment group based on total protein input. Samples were treated with PBS, RNase A, Triton X-100, or RNase A plus Triton X-100. RNase A and Triton X-100 were obtained from Proteinbio (Nanjing, China) and used at final concentrations of 100 μg/mL and 0.1%, respectively. After incubation at 37 °C for 30 min, RNA was extracted using TRIzol reagent, and LINC02701 abundance was measured by qPCR. Relative LINC02701 levels were calculated with reference to the untreated vesicle group.

### 2.5 Granulosa cell culture and exosome treatment

Human ovarian granulosa SVOG cells were cultured in RPMI 1640 medium supplemented with 10% fetal bovine serum. For exosome-transfer experiments, cells were treated with exosomes at a final concentration of 20 μg/mL, normalized by total exosomal protein content for 48 h. Untreated cells served as negative controls. LINC02701 uptake was assessed by qPCR.

### 2.6 Lentiviral overexpression of LINC02701

LINC02701 overexpression (OE) and empty-vector (Vector) lentiviral constructs were introduced into SVOG cells at a multiplicity of infection of 10. Successful transduction was confirmed by ZsGreen fluorescence. Stable lines were generated with puromycin selection (2 μg/mL, 72 h). Overexpression efficiency was confirmed by qPCR.

### 2.7 siRNA-mediated knockdown of LINC02701

To investigate the functional role of endogenous LINC02701, SVOG cells were transfected with small interfering RNA targeting LINC02701 (siLINC02701) or a negative control siRNA (siNC). The siRNAs were designed and synthesized by Proteinbio (Nanjing, China). Transfection was performed using Micropro-transfecter Cell Reagent (Proteinbio, Nanjing, China) according to the manufacturer’s instructions. The final concentration of siRNA used for transfection was 60 nM. Cells were harvested 48 h after transfection for RNA extraction, apoptosis assays, proliferation assays, and Western blot analysis. Knockdown efficiency was assessed by qPCR.

### 2.8 Pharmacological inhibition of P53

To determine whether the pro-apoptotic effect of LINC02701 is mediated through P53 signaling, SVOG cells overexpressing LINC02701 were treated with the P53 inhibitor Pifithrin-α hydrobromide (MedChemExpress, China). Pifithrin-α hydrobromide was dissolved in DMSO to prepare the stock solution and further diluted in complete culture medium to the desired working concentration. Cells were divided into three groups: WT + vehicle, OE + vehicle, and OE + Pifithrin-α. Vehicle-treated cells received an equal volume of DMSO as the solvent control. The final concentration of DMSO in all groups was kept below 0.1%. Cells in the inhibitor group were treated with Pifithrin-α hydrobromide at a final concentration of 10 μM for 24 h, while vehicle groups received the same volume of DMSO. After treatment, cells were collected for apoptosis analysis by Annexin V-APC/7-AAD flow cytometry and for Western blot analysis of apoptosis-related proteins, including BAX, PUMA, and cleaved caspase-3. All experiments were performed in at least three independent biological replicates.

### 2.9 Apoptosis and cell proliferation assays

Apoptosis was quantified using Annexin V-APC/7-AAD (Liankebio, Hangzhou, China) staining followed by flow cytometry. Proliferation was assessed using the CCK-8 assay according to the manufacturer’s protocol (Vazyme, Nanjing, China). These assays were performed in overexpression, knockdown, and rescue experiments as indicated. All experiments were performed in biological triplicate.

### 2.10 RNA pull-down and RNA immunoprecipitation (RIP)

Biotin-labeled LINC02701 sense and antisense RNAs were synthesized *in vitro* and incubated with SVOG lysates. Streptavidin magnetic beads were used to capture RNA–protein complexes, followed by mass spectrometric identification of bound proteins (Guangzhou Fitgene Biotechnology Co., Ltd.). For RIP assays, SVOG cell lysates were incubated with an anti-GRP75 antibody (Proteintech #14887-1-AP, 4 μg antibody per 1–3 mg total protein lysate) or the corresponding normal IgG as a negative control. An aliquot of total lysate was retained as the input control, representing 10% of the total lysate used for immunoprecipitation. Immunoprecipitated complexes were collected using Protein A/G magnetic beads, and the enrichment of LINC02701 was analyzed by qPCR. GRP75 enrichment in the immunoprecipitated fraction was verified by Western blotting.

### 2.11 Co-immunoprecipitation (Co-IP) assays

To determine whether LINC02701 modulates the interaction between GRP75 and P53, Co-IP assays were performed using lysates from LINC02701-overexpressing (OE) and untreated (WT) SVOG cells. Equal amounts of protein lysates were incubated with an anti-GRP75 antibody (Proteintech #14887-1-AP, 4 μg antibody per 1–3 mg total protein lysate) or the corresponding normal IgG as a negative control. An aliquot of total lysate was retained as the input control, representing 10% of the total lysate used for immunoprecipitation. Immunoprecipitated complexes were captured using Protein A/G magnetic beads, and the associated P53 protein was detected by Western blotting using an anti-P53 antibody (Proteintech #10442-1-AP, 1:5,000). Relative P53 enrichment in the immunoprecipitated fractions was quantified by densitometric analysis. Input-normalized P53/GRP75 ratios were compared across groups.

### 2.12 Fluorescence in situ hybridization (FISH) and immunofluorescence (IF)

Cells were fixed, permeabilized, and hybridized with a Cy3-labeled LINC02701 probe (Ribobio, Guangzhou, China). Following hybridization, GRP75 or P53 was detected via immunofluorescence using appropriate primary and Alexa Fluor–conjugated secondary antibodies. Nuclei were counterstained with DAPI. Images were acquired using a confocal microscope (THUNDER DMi8, Leica, Germany) under identical acquisition settings for all groups within each experiment, including laser power, detector gain, and exposure conditions. Fluorescence signals were analyzed using ImageJ software. Briefly, the mean fluorescence intensity of the P53 signal in each field was measured after background subtraction, and relative fluorescence intensity was calculated under identical analysis parameters across groups. Quantification was performed using four random fields per condition in three independent experiments.

### 2.13 RNA extraction and quantitative PCR

Total RNA was extracted using TRIzol reagent (Invitrogen) and reverse-transcribed with HiScript IV (Vazyme #R433). qPCR was performed using SYBR Green Master Mix (Vazyme #Q312) on a StepOne system (Applied Biosystems). For cellular RNA, gene expression was normalized to *ACTB* using the 2^−ΔΔCt^ method. For exosomal RNA, equal amounts of exosome preparations were used as input for RNA extraction based on total exosomal protein content. Because no validated endogenous reference gene or exogenous spike-in control was used in the present study, exosomal LINC02701 levels were quantified under identical processing conditions and expressed relative to the corresponding control group. The primer sequences used in this study are listed in Table 1.

**TABLE 1:** Sequences of primers used.

Gene	Forward primer	Reverse primer
*LINC02701*	5′-GCA​AGA​GCA​GAA​CTC​AGG​AA-3′	5′-ATC​CAC​GTC​CTC​TCA​CAT​TGC-3′
*BAX*	5′- GAT​GGA​CGG​GTC​CGG​GG-3′	5′- CGA​TCC​TGG​ATG​AAA​CCC​TGA-3′
*PUMA*	5′- ATG​AAA​TTT​GGC​ATG​GGG​TCT​G-3′	5′- GCT​CCC​TGG​GGC​CAC​AAA-3′
*BCL2*	5′- AAA​AAT​ACA​ACA​TCA​CAG​AGG​AAG​T-3′	5′- GTT​CCA​CAA​AGG​CAT​CCC​AG-3′
*P21*	5′- AGT​CAG​TTC​CTT​GTG​GAG​CC-3′	5′- AGG​AGA​ACA​CGG​GAT​GAG​GA-3′
*ACTB*	5′- CCA​CCA​TGT​ACC​CTG​GCA​TT-3′	5′- GTC​CTC​GGC​CAC​ATT​GTG​AA-3′

### 2.14 Western blotting

Cells were lysed in RIPA buffer (Epizyme, Shanghai, China) containing protease inhibitors (Epizyme, Shanghai, China). Protein concentration was determined by BCA assay (Epizyme, Shanghai, China). Samples were separated via SDS-PAGE gels (ACE, Changzhou, China) and transferred to PVDF membranes (Merck). Primary antibodies targeted GRP75 (Proteintech #14887-1-AP, 1:5,000); P53 (Proteintech #10442-1-AP, 1:5,000); BAX (Proteintech #50599-2-Ig, 1:20,000); PUMA (Epizyme #R014363, 1:2,000); P21 (Proteintech #82669-2-RR, 1:2,000); Cleaved Caspase-3 (Biodragon #RM8539, 1:2,000); GAPDH (Bioworld #AP0063, 1:5,000). HRP-conjugated secondary antibodies and ECL reagents (ProteinBio, Nanjing, China) were used for detection. Densitometry analysis was performed using ImageJ. All primary antibodies used in this study were commercial antibodies with manufacturer-provided validation for the indicated applications. Antibody specificity in the present study was further supported by detection of bands at the expected molecular weights in input lysates and by the absence of specific enrichment in the corresponding IgG control groups.

### 2.15 Statistical analysis

Data are expressed as mean ± SD. Statistical analysis and graphing were performed using GraphPad Prism 10. Image area, band color, and fluorescence intensity were analyzed using ImageJ. Statistical analysis was performed using SPSS 27. Intergroup comparisons were analyzed using one-way ANOVA with Tukey’s *post hoc* testing or Student’s t-test where appropriate. Statistical significance was set at p < 0.05.

## 3 Results

### 3.1 Clinical characteristics of participants

Analysis of baseline clinical parameters demonstrated clear distinctions among the three groups (Table 2). Serum CA125 levels were significantly higher in the EMs_A group compared with both the EMs_C and Ctrl groups (p < 0.0001), reflecting a state of active inflammation consistent with previous reports of elevated CA125 during disease exacerbation (Hirsch et al., 2017). However, CA125 is not specific to endometriosis and should be interpreted in conjunction with other clinical features. Uterosacral ligament tenderness scores also differed significantly across the groups (p < 0.0001), with EMs_A patients exhibiting the highest levels of pain sensitivity. Importantly, Day-3 high-quality embryo rates were markedly reduced in EMs_A compared with Ctrl patients (p < 0.05), suggesting impaired early embryo quality in the context of active endometriosis. Day-3 high-quality embryos were defined as cleavage-stage embryos graded I or II on Day 3 after fertilization, and the Day-3 high-quality embryo rate was calculated as the number of grade I or II embryos divided by the number of 2 PN fertilized embryos, according to the morphological embryo grading system routinely used in our center and consistent with established cleavage-stage embryo assessment criteria (Alpha Scientists in Reproductive Medicine and ESHRE Special Interest Group of Embryology, 2011). These clinical observations provided the rationale for further investigation into exosomal cargo alterations and their functional consequences.

**TABLE 2:** The general condition and IVF-related indices of the enrolled patients.

Items	Ctrl	EMs_C	EMs_A	F	p
n	8	9	7	​	​
Age (years)	30.38 ± 2.67	29.33 ± 2.29	31.00 ± 3.27	0.772	0.4748
BMI (kg/m^2^)	21.41 ± 1.85	21.22 ± 2.47	20.67 ± 2.24	0.224	0.8013
AMH (ng/mL)	5.53 ± 2.51	3.97 ± 1.45	3.81 ± 2.05	1.736	0.2005
Antral follicles, n	15.25 ± 4.27	15.78 ± 4.18	13.14 ± 6.91	0.558	0.5808
VAS score for ULT	0.00 ± 0.00^a^	3.28 ± 0.57^b^	6.86 ± 0.75^c^	311.796	**<0.0001**
CA125 (U/mL)	9.67 ± 1.04^a^	9.86 ± 3.20^a^	35.18 ± 12.80^b^	23.326	**<0.0001**
Follicles with size ≥14 mm	11.25 ± 2.82	12.44 ± 3.47	8.57 ± 4.04	2.542	0.1026
Retrieved oocytes, n	13.13 ± 4.36	13.33 ± 2.87	9.14 ± 5.82	2.175	0.1385
Fertilization rate (%)	85.53 ± 14.01	77.83 ± 16.02	69.97 ± 15.79	1.932	0.1698
D3 high-quality embryos (%)	74.60 ± 14.79^a^	57.60 ± 28.39^ab^	45.07 ± 14.71^b^	3.751	**0.0405**
Blastocyst rate (%)	43.91 ± 23.40	43.50 ± 33.97	69.19 ± 45.71	0.815	0.4613

Ctrl, control group patients; EMs_C, patients with well-controlled endometriosis; EMs_A, patients with active endometriosis; BMI, body mass index; AMH, anti-Müllerian hormone; CA125, carbohydrate antigen 125; D3, the third day; ULT, uterosacral ligament tenderness; Fertilization rate = number of two-pronuclear (2 PN) fertilized embryos/MII, eggs; MII, metaphase II; D3 high-quality embryo rate = number of embryos graded I or II, on Day 3 after fertilization/number of 2 PN, fertilized embryos.

a, b, c The difference between the two groups with different letters superscript was statistically significant.

All p < 0.05 are highlighted in bold.

### 3.2 Characterization of follicular fluid-derived exosomes

**FIGURE 1:**
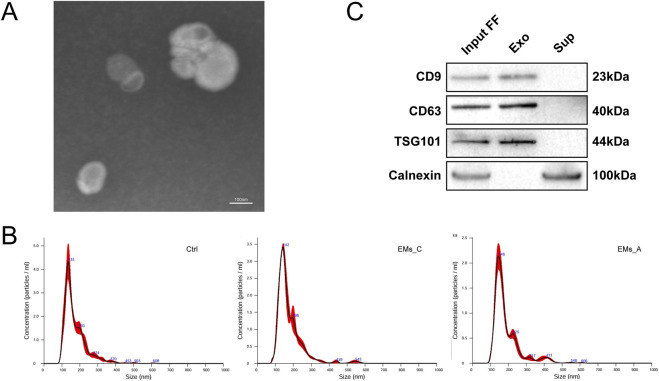
Characterization of follicular fluid-derived exosomes. **(A)** Transmission electron microscopy (TEM) showing a representative field containing multiple extracellular vesicles with typical exosome-like morphology. **(B)** Nanoparticle tracking analysis (NTA) showing the size distribution of vesicles isolated from the Ctrl, EMs_C, and EMs_A groups. **(C)** Western blot analysis of the input follicular fluid (Input FF), exosome pellet (Exo), and exosome-depleted supernatant (Sup) fractions. Canonical exosomal markers (CD9, CD63, and TSG101) were enriched in the exosome pellet, whereas the endoplasmic reticulum marker Calnexin was absent in the exosome pellet.

Transmission electron microscopy revealed a representative field containing multiple extracellular vesicles with typical exosome-like morphology (Figure 1A). Nanoparticle tracking analysis showed that vesicles isolated from the Ctrl, EMs_C, and EMs_A groups exhibited comparable size distributions, with the majority of particles ranging from approximately 100–200 nm in diameter (Figure 1B). Western blot analysis using input follicular fluid (Input FF), exosome pellet (Exo), and exosome-depleted supernatant (Sup) demonstrated enrichment of the canonical exosomal markers CD9, CD63, and TSG101 in the exosome pellet fraction. In contrast, the endoplasmic reticulum marker Calnexin was detected in the non-exosomal fractions but was absent in the exosome pellet (Figure 1C). These findings support the identity of the isolated vesicles and indicate minimal contamination from intracellular components.

### 3.3 Exosomal LINC02701 is markedly upregulated in active endometriosis

**FIGURE 2:**
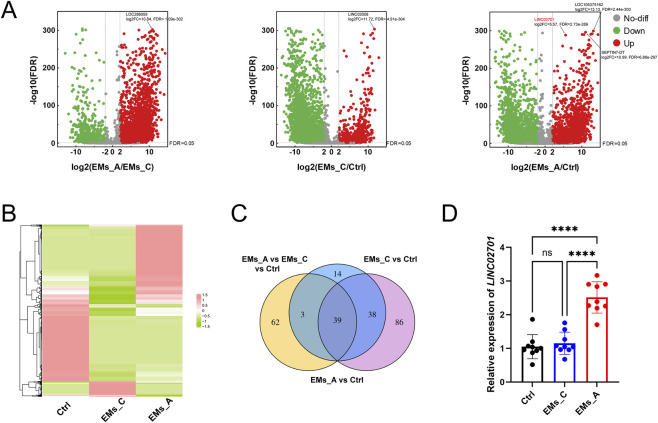
Identification and validation of LINC02701 as a key exosomal lncRNA in endometriosis. **(A)** Volcano plots of differentially expressed lncRNAs. Up, significantly upregulated lncRNAs (log2FC > 2, FDR <0.05); Down, significantly downregulated lncRNAs (log2FC < −2, FDR <0.05); No-diff, non-differentially expressed lncRNAs. Labels of representative top lncRNAs indicate the exact log2FC and FDR values. **(B)** Heatmap of lncRNA expression across groups. Color scale, green (low) to red (high) expression. **(C)** Venn diagram of candidate lncRNAs. Set 1: lncRNAs with log2(EMs_A/Ctrl) > 2, Set 2: lncRNAs with log2(EMs_C/Ctrl) > 2, Set 3: lncRNAs showing expression gradient: Ctrl < EMs_C < EMs_A; Core: 39 candidate lncRNAs (including LINC02701). **(D)** qPCR validation of LINC02701 expression in exosomes. ****p < 0.0001; Error bars represent mean ± SD.

High-throughput sequencing of exosomal RNA revealed substantial transcriptomic differences among the three groups. LINC02701 emerged as one of the most significantly upregulated lncRNAs in exosomes from EMs_A compared with Ctrl (log_2_FC = 5.57, FDR = 2.73 × 10^-289) (Figures 2A–C). In contrast, its expression was lower in the EMs_C group, indicating an association with disease activity. qPCR validation confirmed this pattern (Figure 2D), supporting LINC02701 as a disease activity-associated exosomal lncRNA in endometriosis. This finding is consistent with recent evidence indicating that exosome-incorporated lncRNAs can reflect the metabolic state of the follicular microenvironment (Liu et al., 2024; Zhang et al., 2025).

### 3.4 Exosomal LINC02701 is protected within membrane-enclosed vesicles and efficiently delivered to granulosa cells

**FIGURE 3:**
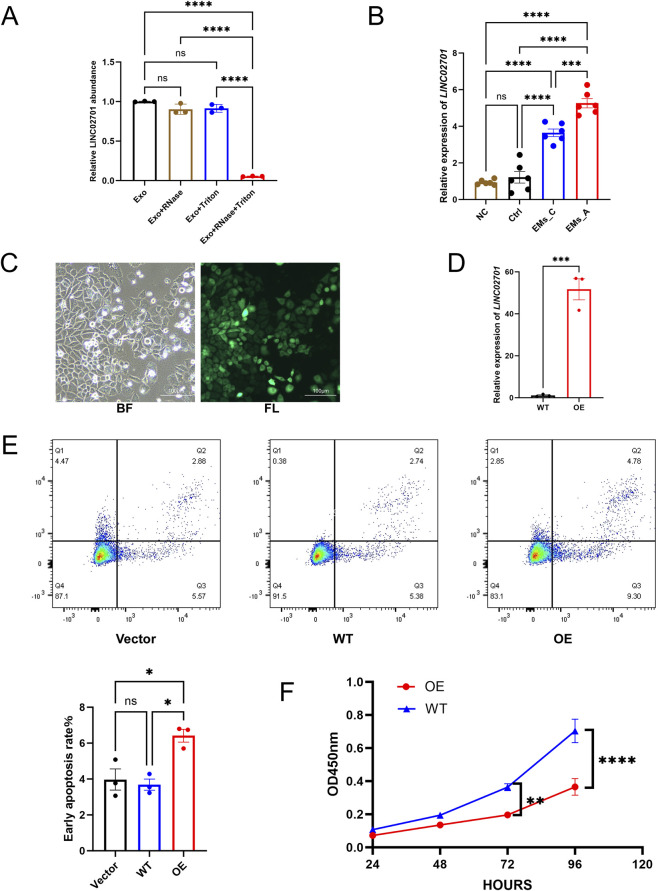
Vesicle-associated LINC02701 is transferred to granulosa cells, and LINC02701 overexpression promotes apoptosis and suppresses proliferation. **(A)** RNase protection assay showing that RNase treatment alone did not significantly reduce LINC02701 abundance, whereas combined RNase and Triton X-100 treatment markedly decreased LINC02701 levels, indicating that LINC02701 is protected within membrane-enclosed vesicles. **(B)** Relative expression of LINC02701 in SVOG cells after co-culture with exosomes for 48 h. NC, equal volume of PBS. **(C)** Bright-field (BF) and fluorescence (FL) images of lentivirus-transduced SVOG cells. Scale bar = 100 μm. **(D)** qPCR validation of LINC02701 overexpression efficiency. **(E)** Representative flow cytometry plots and quantification of early apoptotic cells. **(F)** CCK-8 assay showing cell proliferation. Vector, empty vector control cells; WT, untreated cells; OE, LINC02701-overexpressing cells. Error bars represent mean ± SD. ns, not significant; *p < 0.05, **p < 0.01, ***p < 0.001, ****p < 0.0001.

To determine whether LINC02701 was protected within membrane-enclosed vesicles, purified exosomes were subjected to RNase protection assays. qPCR analysis showed that RNase treatment alone did not significantly reduce LINC02701 abundance, whereas combined RNase and detergent treatment markedly decreased LINC02701 levels, indicating that LINC02701 was protected within membrane-enclosed vesicles (Figure 3A). To further assess whether exosomal LINC02701 could be transferred to granulosa cells, SVOG cells were co-cultured with exosomes derived from different groups. Intracellular LINC02701 levels were significantly increased after exposure to exosomes from the EMs_A group compared with the Ctrl and EMs_C groups (Figure 3B), supporting efficient transfer of exosomal LINC02701 into recipient granulosa cells. This observation is consistent with the established role of EVs in mediating RNA transfer within the ovarian follicle (Varik et al., 2025).

### 3.5 LINC02701 overexpression promotes granulosa cell apoptosis and Inhibits proliferation

To determine whether elevated LINC02701 influences granulosa-cell function, SVOG cells were engineered to stably overexpress the transcript (Figures 3C,D). Flow cytometry revealed a significant increase in early apoptosis in LINC02701-OE cells compared with Vector and WT controls (p < 0.05; Figure 3E). CCK-8 assays demonstrated that LINC02701-OE cells exhibited markedly reduced proliferation (p < 0.0001; Figure 3F). These functional changes parallel the diminished embryo quality observed in EMs_A patients and align with evidence linking granulosa-cell apoptosis to adverse reproductive outcomes (Høst et al., 2002; Lee et al., 2001; Voros et al., 2025).

### 3.6 LINC02701 directly interacts with GRP75

**FIGURE 4:**
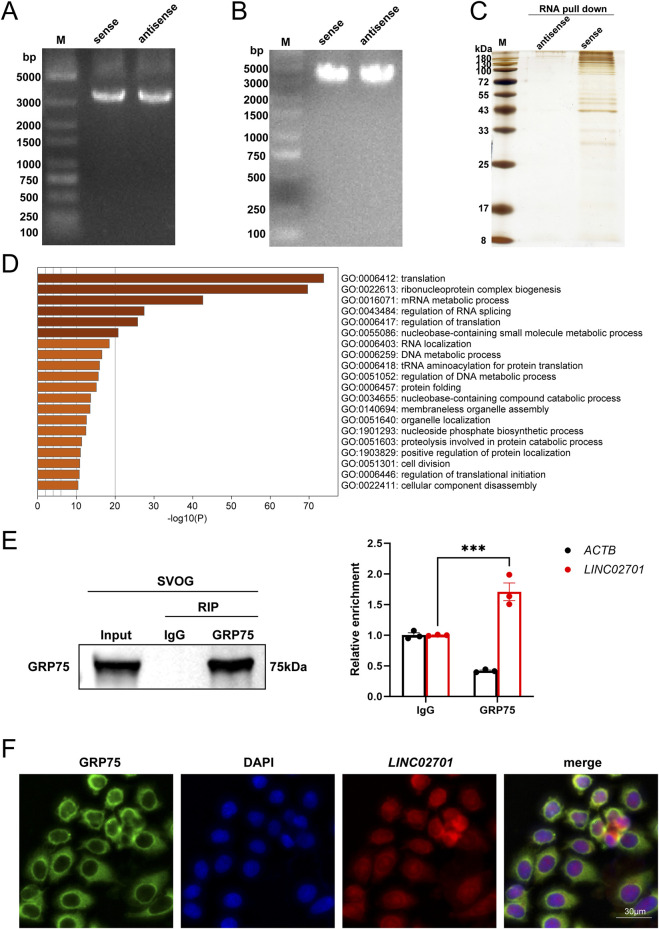
Mechanistic validation of LINC02701–GRP75 interaction. **(A–C)** RNA pull-down assay. **(A)** PCR validation of LINC02701 template. **(B)** Biotinylated RNA product validation. **(C)** SDS-PAGE silver staining of RNA pull-down eluates. **(D)** Mass spectrometry and GO enrichment analysis of proteins associated with LINC02701 (Biological Processes). **(E)** RNA immunoprecipitation (RIP) assay performed with an anti-GRP75 antibody or IgG control. GRP75 enrichment in the immunoprecipitated fraction was confirmed by Western blotting, with 10% Input and IgG as controls. LINC02701 enrichment in the RIP products was subsequently quantified by qPCR. **(F)** FISH-IF analysis showing the subcellular localization of LINC02701 and GRP75. Scale bar = 30 μm.

RNA pull-down assays followed by mass spectrometry identified heat shock protein family A (Hsp70) member 9 (HSPA9/GRP75) as a major LINC02701-associated protein, with a peptide coverage of 29.75% (Figures 4A–D). RIP-qPCR further confirmed that LINC02701 was significantly enriched in GRP75 immunoprecipitates compared to IgG controls (p < 0.001; Figure 4E), confirming a direct and specific interaction. FISH-IF analysis showed predominant cytoplasmic colocalization of LINC02701 and GRP75 (Figure 4F), supporting the spatial feasibility of this molecular association. These data align with current models suggesting that lncRNAs can modulate stress-response pathways by interacting with mitochondrial chaperones such as GRP75 (Yuan et al., 2022).

### 3.7 LINC02701 weakens the GRP75–P53 interaction and triggers apoptotic signaling

**FIGURE 5:**
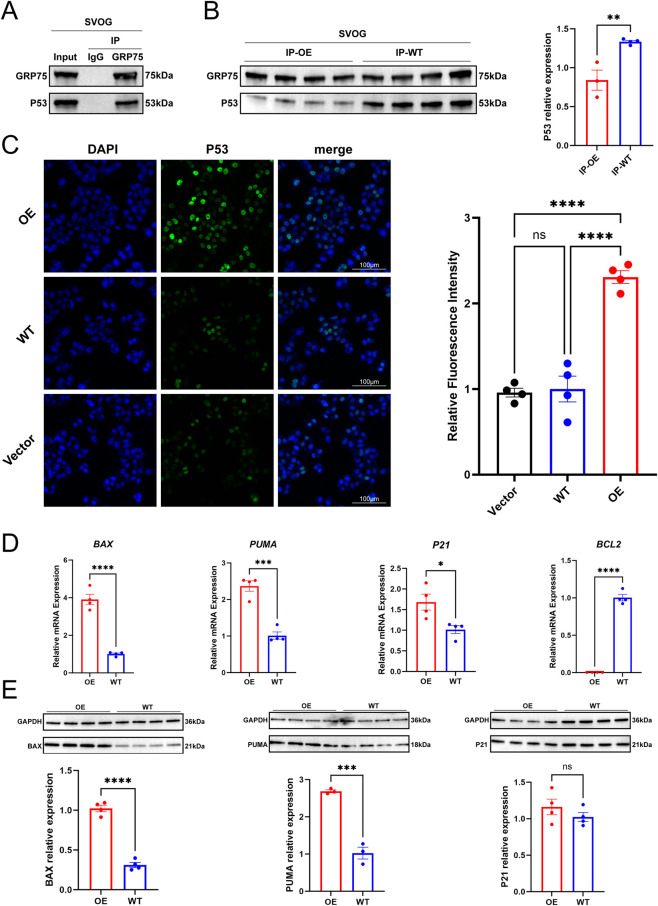
LINC02701 weakens the GRP75–P53 interaction and enhances P53-associated apoptotic signaling. **(A)** Co-immunoprecipitation (Co-IP) assay validating the interaction between GRP75 and P53 in SVOG cells, with Input, IgG, and GRP75 IP controls shown. **(B)** Co-IP analysis of the GRP75–P53 interaction in LINC02701-overexpressing (OE) and untreated (WT) SVOG cells, with quantitative analysis of relative P53 expression in the immunoprecipitated fractions. **(C)** Immunofluorescence staining of P53 in Vector, WT, and OE cells, together with quantitative analysis of relative fluorescence intensity. Relative P53 fluorescence intensity was quantified using ImageJ after background subtraction from four random fields per condition in three independent experiments. Scale bar = 100 μm. **(D)** qPCR analysis of apoptosis-related genes, including *BAX*, *PUMA*, *P21*, and *BCL2*, in OE and WT cells. **(E)** Western blot analysis of BAX, PUMA, and P21 protein expression in OE and WT cells, together with densitometric quantification normalized to GAPDH. OE, LINC02701-overexpressing cells; WT, untreated cells; Vector, empty vector control cells; IP-OE, immunoprecipitated fraction from OE cells; IP-WT, immunoprecipitated fraction from WT cells. Error bars represent mean ± SD. *p < 0.05, **p < 0.01, ***p < 0.001, ****p < 0.0001; ns, not significant.

Because GRP75 is known to regulate P53 localization and activity, we examined whether LINC02701 affects this interaction. Co-IP assays demonstrated that GRP75–P53 binding was markedly reduced in LINC02701-OE cells compared with WT cells (Figures 5A,B). This disruption correlated with increased nuclear accumulation of P53, as shown by IF quantification (Figure 5C). Western blotting further revealed that LINC02701 overexpression led to upregulation of downstream P53 targets, including BAX, PUMA, and P21 (Figures 5D,E), all key mediators of apoptotic execution. These effects closely mirror the apoptotic phenotype induced by exosomes from EMs_A patients.

### 3.8 LINC02701 knockdown attenuates granulosa cell apoptosis

**FIGURE 6:**
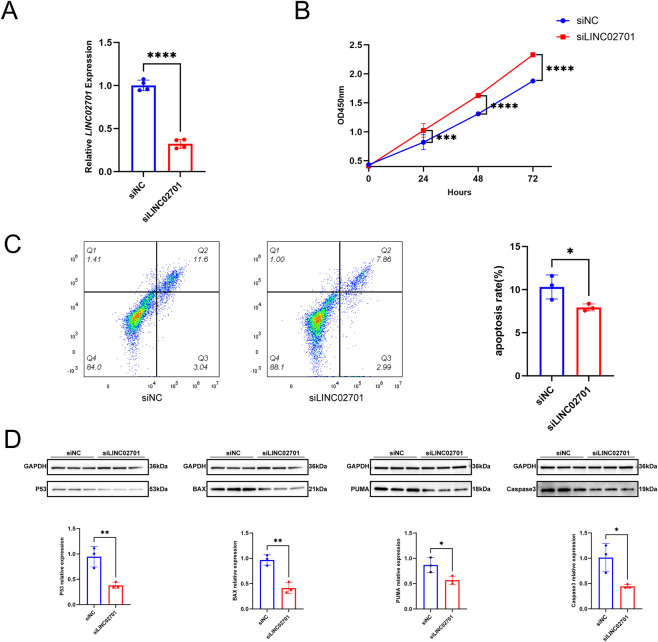
LINC02701 knockdown attenuates granulosa cell apoptosis and restores proliferation. **(A)** qPCR validation of knockdown efficiency. **(B)** CCK-8 proliferation assay. **(C)** Representative flow cytometry plots and quantification of apoptotic cells. **(D)** Western blot analysis of apoptosis-related proteins and densitometric quantification of Western blot results. siNC, negative control siRNA; siLINC02701, LINC02701-targeting siRNA. Error bars represent mean ± SD.

To determine whether endogenous LINC02701 is required for granulosa cell apoptosis, SVOG cells were transfected with siLINC02701 or control siRNA. qPCR analysis showed that transfection with siLINC02701 significantly reduced LINC02701 expression compared with the siNC group (p < 0.0001; Figure 6A), confirming effective knockdown. Flow cytometry analysis demonstrated that knockdown of LINC02701 significantly reduced granulosa cell apoptosis (Figure 6C). Consistent with these findings, CCK-8 assays showed that LINC02701 knockdown significantly enhanced cell proliferation compared with the control group (Figure 6B). Furthermore, Western blot analysis demonstrated that knockdown of LINC02701 markedly reduced the expression of P53 downstream pro-apoptotic proteins, including BAX, PUMA, and cleaved caspase-3 (Figure 6D), suggesting that endogenous LINC02701 contributes to granulosa cell apoptosis.

### 3.9 Inhibition of P53 attenuates LINC02701-induced apoptosis

**FIGURE 7:**
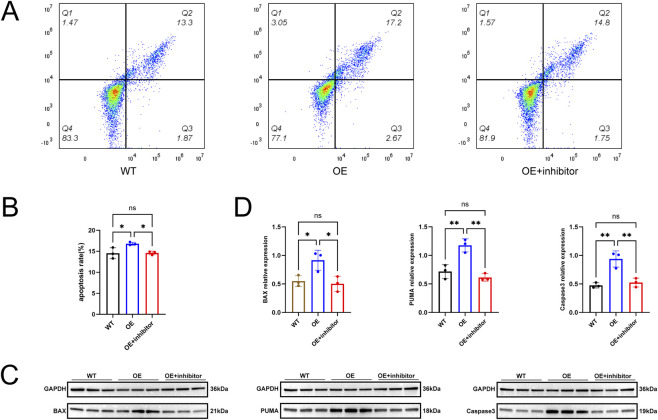
Inhibition of P53 attenuates LINC02701-induced apoptosis. **(A)** Representative flow cytometry plots of apoptosis in WT + vehicle, OE + vehicle, and OE + Pifithrin-α groups. **(B)** Quantification of apoptotic cells. **(C)** Western blot analysis of apoptosis-related proteins, including BAX, PUMA, and cleaved caspase-3. **(D)** Densitometric quantification of Western blot results. WT, untreated cells; OE, LINC02701-overexpressing cells. Error bars represent mean ± SD.

To further determine whether the pro-apoptotic effect of LINC02701 is mediated through P53 signaling, SVOG cells overexpressing LINC02701 were treated with the P53 inhibitor Pifithrin-α. Flow cytometry analysis showed that LINC02701 overexpression increased apoptosis in SVOG cells (16.8% ± 0.36%) compared with WT cells (14.53% ± 1.31%). Treatment with Pifithrin-α partially reduced apoptosis to 14.6% ± 0.44% (p < 0.05) (Figures 7A,B), indicating that the pro-apoptotic effect of LINC02701 is at least partially dependent on P53 activation. Consistently, Western blot analysis demonstrated that inhibition of P53 significantly attenuated the upregulation of BAX, PUMA, and cleaved caspase-3 induced by LINC02701 overexpression (Figures 7C,D).

Taken together, these findings support the involvement of the GRP75–P53 axis in LINC02701-induced granulosa cell apoptosis.

## 4 Discussion

### 4.1 Principal findings

This study identifies exosomal LINC02701 as a disease activity-dependent regulator of granulosa-cell function in endometriosis. LINC02701 was markedly enriched in follicular fluid-derived exosomes from women with active endometriosis and was efficiently transferred into granulosa cells. Functionally, in SVOG cells, LINC02701 overexpression suppressed granulosa-cell proliferation and promoted apoptosis, whereas knockdown of LINC02701 attenuated apoptosis and partially restored cell proliferation. Mechanistically, it directly interacted with GRP75, weakened the GRP75–P53 interaction, facilitated P53 nuclear accumulation, and activated downstream apoptotic targets, including BAX and PUMA. Clinically, this molecular dysfunction was accompanied by significantly elevated CA125 levels, increased uterosacral ligament tenderness, and reduced Day-3 high-quality embryo rates in EMs_A patients. The first two are clinical manifestations of active endometriosis, while the decline in embryo quality represents an early embryonic manifestation of the adverse reproductive effects of active disease. Together, these findings support a link between microenvironmental inflammation, exosomal cargo alteration, granulosa-cell injury, and impaired early embryo quality in active endometriosis.

### 4.2 Integration of clinical and molecular evidence

The clinical features observed in patients with active endometriosis are broadly consistent with the molecular findings of this study and support a link between inflammatory disease activity and altered exosomal signaling. In the present cohort, CA125 levels were significantly elevated in the EMs_A group. Although CA125 has been associated with inflammatory activity in endometriosis, it is not specific to this disease and may also be influenced by other gynecological or inflammatory conditions; therefore, it should be interpreted in combination with the broader clinical context rather than as an endometriosis-specific marker (Hirsch et al., 2017). By contrast, increased uterosacral ligament tenderness more directly reflects localized pelvic inflammation and nociceptive sensitization, both of which are characteristic of active disease (Cetera et al., 2023; Shafrir et al., 2021). Such inflammatory conditions have been shown to remodel the follicular microenvironment and alter EV biogenesis, cargo selection, and downstream signaling (Ding et al., 2025; Xiao et al., 2022). In parallel with these clinical indicators, the reduced Day-3 high-quality embryo rate observed in the EMs_A group suggests impaired early embryo quality in the setting of active endometriosis and is consistent with previous studies reporting compromised early embryo development in affected women (Cohen et al., 2015). Our functional data provide a plausible mechanistic explanation for this association. Specifically, exosomal LINC02701 promoted granulosa-cell apoptosis and suppressed proliferation, whereas knockdown of LINC02701 attenuated these effects. Given the central role of granulosa cells in supporting oocyte metabolism, survival, and meiotic progression, disruption of granulosa-cell homeostasis would be expected to adversely affect the developmental environment of the oocyte (Hussein, 2005; Lee et al., 2001). Taken together, the concordance between the clinical phenotype and the *in vitro* findings supports the biological relevance of LINC02701 as a disease activity-associated exosomal factor in endometriosis-related reproductive dysfunction.

### 4.3 Biological implications

**FIGURE 8:**
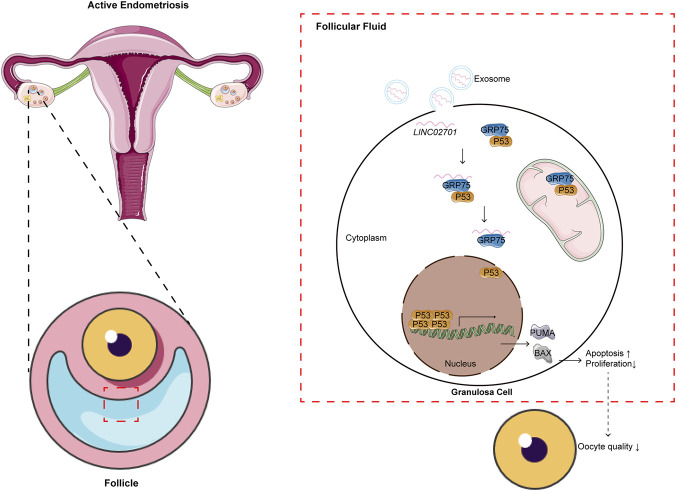
Proposed mechanism by which follicular fluid-derived exosomal LINC02701 promotes granulosa-cell apoptosis in active endometriosis. Exosomal LINC02701 enters granulosa cells, interacts with GRP75, weakens the GRP75–P53 interaction, promotes P53 accumulation, and activates downstream apoptotic signaling.

Emerging evidence highlights the crucial role of extracellular vesicles in shaping the ovarian microenvironment and mediating intercellular RNA exchange (Hung et al., 2015; Varik et al., 2025). Our findings extend this concept by identifying LINC02701 as a vesicle-enriched lncRNA associated with granulosa-cell apoptosis in active endometriosis. Mechanistically, the observation that LINC02701 directly interacts with GRP75 and weakens its interaction with P53 is consistent with current understanding of mitochondrial–nuclear stress communication (Yuan et al., 2022). GRP75 acts as a molecular chaperone that sequesters P53 in the cytoplasm and prevents aberrant activation of pro-apoptotic pathways (Kaul et al., 2005). In the present study, disruption of this protective interaction was associated with increased P53 accumulation and upregulation of downstream apoptotic factors, including BAX, PUMA, P21, and cleaved caspase-3. Moreover, the rescue experiments using a P53 inhibitor further support that the pro-apoptotic effect of LINC02701 is at least partially dependent on P53 signaling. Collectively, these findings support a model in which active endometriosis-associated inflammation promotes granulosa-cell apoptosis, at least in part, through modulation of the LINC02701–GRP75–P53 axis (Figure 8).

An additional biological question raised by the present findings is how LINC02701 becomes selectively enriched in extracellular vesicles. Increasing evidence suggests that extracellular RNA loading is not merely passive, but may be regulated by multiple mechanisms, including recognition by RNA-binding proteins, sequence- or structure-dependent sorting, and vesicle biogenesis pathways responsive to cellular stress or inflammation. In this context, the inflammatory milieu of active endometriosis may influence not only the expression of LINC02701 itself, but also its preferential incorporation into extracellular vesicles. Although the current study did not directly examine the sorting machinery responsible for LINC02701 packaging, these observations raise the possibility that selective vesicular loading may represent an additional regulatory layer linking inflammatory activity to granulosa-cell dysfunction. Further studies will be needed to determine whether specific RNA-binding proteins or vesicle biogenesis pathways contribute to LINC02701 enrichment in extracellular vesicles in active endometriosis.

### 4.4 Comparison with previous studies

Most previous studies of extracellular vesicle-associated regulatory molecules in reproductive biology have primarily focused on microRNAs, whereas relatively few have examined the role of lncRNAs, particularly in the context of endometriosis. Even fewer studies have addressed whether exosomal lncRNA alterations are associated with disease activity rather than the mere presence of disease. In this regard, the identification of LINC02701 as a vesicle-enriched lncRNA associated with active endometriosis is consistent with recent reports showing substantial remodeling of the follicular microenvironment in affected women (Wei et al., 2023). Our findings also complement accumulating evidence that non-coding RNAs can influence mitochondrial function, cellular stress responses, and survival signaling in ovarian cells. By linking a follicular fluid-derived exosomal lncRNA to GRP75–P53-associated apoptotic signaling, the present study extends current understanding beyond descriptive exosomal profiling and provides a more mechanistic framework for how altered vesicle cargo may contribute to granulosa-cell dysfunction in endometriosis. At the same time, our study adds to the emerging concept that the biological effects of extracellular vesicle cargo in reproductive disorders may depend not only on the identity of the molecule itself, but also on the inflammatory and endocrine context in which it is produced and transferred.

### 4.5 Clinical implications

The combined clinical, cellular, and molecular findings suggest several potential translational implications. First, because LINC02701 expression was highest in EMs_A and was associated with clinical indicators of disease activity, it may represent a candidate biomarker for evaluating inflammatory status in EMs. However, this possibility requires validation in larger independent cohorts before any predictive application can be inferred. Exosome-based biomarkers are increasingly recognized for their diagnostic value in reproductive diseases (Voros et al., 2025). Second, mechanistic insights into the LINC02701–GRP75–P53 axis suggest that this pathway may represent a potential therapeutic direction for future investigation aimed at restoring granulosa cell viability; however, validation in primary granulosa cells and more physiologically relevant models will be required before translational implications can be inferred. Third, given the reduced Day-3 embryo quality observed in EMs_A patients, further studies are warranted to determine whether exosomal lncRNA profiles have value in clinical stratification or treatment optimization.

### 4.6 Strengths and limitations

Strengths of this study include its integrated design, which combines clinical observations, exosomal transcriptomic profiling, cellular functional assays, and mechanistic biochemical analyses to construct a multi-level model linking endometriosis-associated inflammation to granulosa-cell dysfunction. The identification of LINC02701 as a disease activity-associated vesicle-enriched lncRNA, together with gain-of-function, loss-of-function, and rescue experiments, strengthens the mechanistic relevance of the proposed LINC02701–GRP75–P53 axis.

Several limitations should nevertheless be acknowledged. First, the clinical cohort was relatively small and derived from a single center, and heterogeneity in ovarian stimulation protocols may have further contributed to variation in the follicular microenvironment and extracellular vesicle cargo, which may have limited the statistical power for detecting subtle clinical associations and restricts the generalizability of the findings. Therefore, conclusions regarding clinical correlation and biomarker-related implications should be interpreted cautiously, and larger independent, ideally multicenter, cohorts will be required for validation. Second, although vesicle characterization was strengthened in the revised study by adding additional positive and negative markers and by performing RNase protection assays, the ultracentrifugation-based isolation approach does not fully distinguish exosomes from other small extracellular vesicle subtypes. Accordingly, while our data support that LINC02701 is enriched in membrane-enclosed vesicles rather than existing predominantly as free extracellular RNA, the precise vesicle subtype carrying LINC02701 remains to be further defined. Third, the functional experiments were performed in the immortalized SVOG cell line rather than primary granulosa cells. Although SVOG cells are widely used for mechanistic studies, they may not fully recapitulate the biological properties of primary granulosa cells, particularly with respect to vesicle uptake, stress responses, and P53 dynamics. Fourth, although rescue experiments using pharmacological inhibition of P53 were added in the revised study and support the involvement of this pathway, additional mechanistic validation, such as GRP75 overexpression or genetic perturbation approaches, will be required to further define pathway specificity. In addition, exosome treatment was performed using a single working concentration normalized by total exosomal protein content, and dose-response effects were not evaluated in the present study. Finally, the upstream mechanisms governing selective packaging of LINC02701 into extracellular vesicles remain unexplored, including whether this process is mediated by specific RNA-binding proteins, RNA motifs, or vesicle biogenesis pathways.

### 4.7 Future directions

Future studies should focus on several directions. First, larger independent cohorts will be needed to validate the association of exosomal LINC02701 with disease activity and reproductive outcomes in endometriosis. In particular, protocol-matched or protocol-stratified cohorts should be included to better distinguish disease activity-associated changes in exosomal cargo from potential confounding effects related to ovarian stimulation regimens, especially GnRH antagonist versus agonist protocols. Second, the functional effects of exosomal LINC02701 should be confirmed in primary granulosa or cumulus cells obtained from IVF patients to strengthen the physiological relevance of the current findings. Third, further work is required to investigate the inflammatory or hormonal signals that regulate LINC02701 transcription and its selective loading into extracellular vesicles. Finally, more physiologically relevant models will be needed to determine whether disrupting the LINC02701–GRP75 interaction can rescue granulosa-cell viability and improve reproductive outcomes.

## 5 Conclusion

In conclusion, this study demonstrates that exosomal LINC02701 promotes granulosa-cell apoptosis and that this effect is at least partially dependent on P53 signaling, supporting the involvement of the GRP75–P53 axis in active endometriosis. These findings identify LINC02701 as a mechanistically relevant regulator of granulosa-cell dysfunction and suggest that it may serve as a candidate biomarker of disease activity in endometriosis. However, its clinical value and biological significance require further validation in larger and independent cohorts.

## Data Availability

The sequencing datasets presented in this study have been deposited in the Genome Sequence Archive (GSA) repository under accession number PRJCA064384.
